# Supplementary material for: Enhancer RNAs predict enhancer–gene regulatory links and are critical for enhancer function in neuronal systems
Source: Nucleic Acids Res. 2020 Aug 18;48(17):9550–70. doi: 10.1093/nar/gkaa671 (PMC7515708; doi:10.1093/nar/gkaa671)
Supplement: gkaa671_Supplemental_Files [file gkaa671_supplemental_files.zip › Supplementary Files Combined.pdf]

SUPPLEMENTARY FILES

Supplementary Data Table 1. List and locations of all identified TAPEs
Supplementary Data Table 2. List and locations of all identified region-selective TAPEs
Supplementary Data Table 3. List a of all transcription factor binding motifs identified by HOMER analysis
Supplementary Data Table 4. Sequences of primers, guides, ASOs, gRNAs, acRNAs, and smFISH probe sets.
Supplementary Data Table 5. List, locations, and predicted target genes of 96 activity-regulated TAPEs
Supplementary Figure 1. Activity dependence and synthesis of *Fos* eRNAs
Supplementary Figure 2. Cellular localization of *Fos* eRNA and mRNA
Supplementary Figure 3. Enhancer activation increases *Fos* eRNA and mRNA expression
Supplementary Figure 4. CRISPRa selectively activates targeted enhancer and linked gene without altering other enhancers or genes
Supplementary Figure 5. eRNA levels in response to dCas9-HAT targeting to enhancers
Supplementary Methods

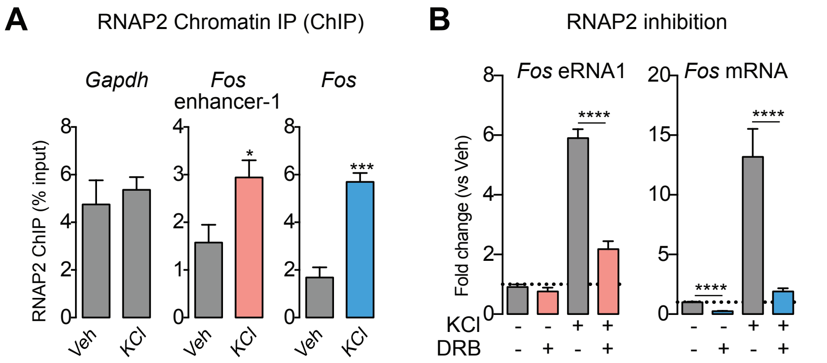

Supplementary Figure 1. Activity dependence and synthesis of *Fos* eRNAs. **A**, RNAP2 ChIP reveals increased recruitment of RNAP2 to the *Fos* enhancer-1 and *Fos* gene body after KCl-mediated depolarization (unpaired t-test, for *Gapdh* promoter region $t(6)$ 0.528, p = 0.6164; *Fos* enhancer-1 $t(6)$ = 2.651, p = 0.038, and *Fos* gene body $t(6)$ = 7.812, p = 0.0004). **B**, 2 hr pre-treatment with RNAP2 dependent transcription inhibitor DRB prior to 1 hr KCl treatment blocked KCl mediated induction of *Fos* eRNA1 and mRNA (two-way ANOVA, for eRNA1 $F(1,42)$ = 27.84 p < 0.0001, and mRNA $F(1,42)$ = 53.42 p < 0.0001, with Tukey's post hoc test for multiple comparison). Data expressed as mean ± s.e.m. Multiple comparisons, *p<0.05, **p<0.01, ***p<0.001, ****p<0.0001.

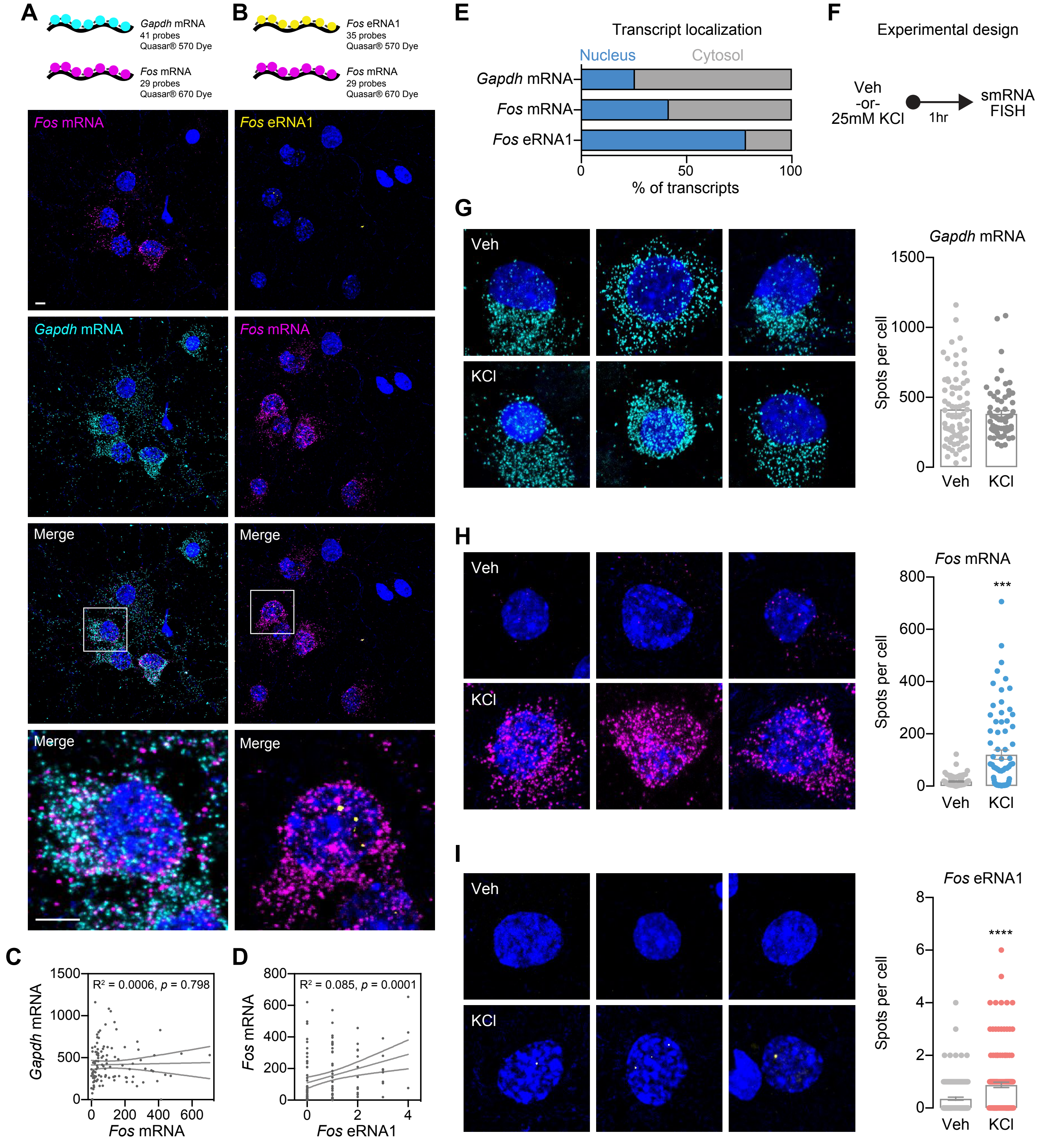

Supplementary Figure 2. Cellular localization of *Fos* eRNA and mRNA. **A-B**, Top panel, illustration of smFISH probe sets indicating number of probes, dye, and LUT. Bottom panel, representative smFISH images for *Gapdh* mRNA (Quasar® 570) and *Fos* mRNA (Quasar® 670) (**A**), and *Fos* eRNA1 (Quasar® 570) and *Fos* mRNA (Quasar® 670) transcripts (**B**). Cell nuclei are stained with DAPI (blue), RNA transcripts are marked by smFISH probes (cyan, magenta, and yellow). Scale bar = 5 μm. **C-D**, Comparison and correlation of detected *Gapdh* mRNA, *Fos* mRNA, and *Fos* eRNA1 spots per cell. While there is no significant correlation between *Gapdh* mRNA and *Fos* mRNA (Pearson correlation for *Gapdh* mRNA and *Fos* mRNA, R^2=0.000586, p=0.7982), *Fos* mRNA and *Fos* eRNA1 are positively correlated on a single cell level (Pearson correlation, R^2=0.08481, p=0.0014). **E**, Compartmentalization of *Gapdh* mRNA, *Fos* mRNA, and *Fos* eRNA1. **F**, Experimental design for neuronal depolarization experiments. **G-I**, Representative images and summary data of *Gapdh* mRNA (top panel), *Fos* mRNA (middle panel), and eRNA1 (bottom panel) after 1 hr of Veh or 25mM KCl treatment. Number of detected *Fos* mRNA and *Fos* eRNA1 transcripts change significantly after stimulation (Mann-Whitney test for Gapdh n(veh)=72, n(KCl)=63, U=2187, p=0.7209; *Fos* mRNA n(veh)=77, n(KCl)=76, U=1929, p=0.0002; eRNA1 n(Veh)=124, n(KCl)=141, U=6540, p<0.0001). Data expressed as mean ± s.e.m. Multiple comparisons, *p<0.05, **p<0.01, ***p<0.001, ****p<0.0001.

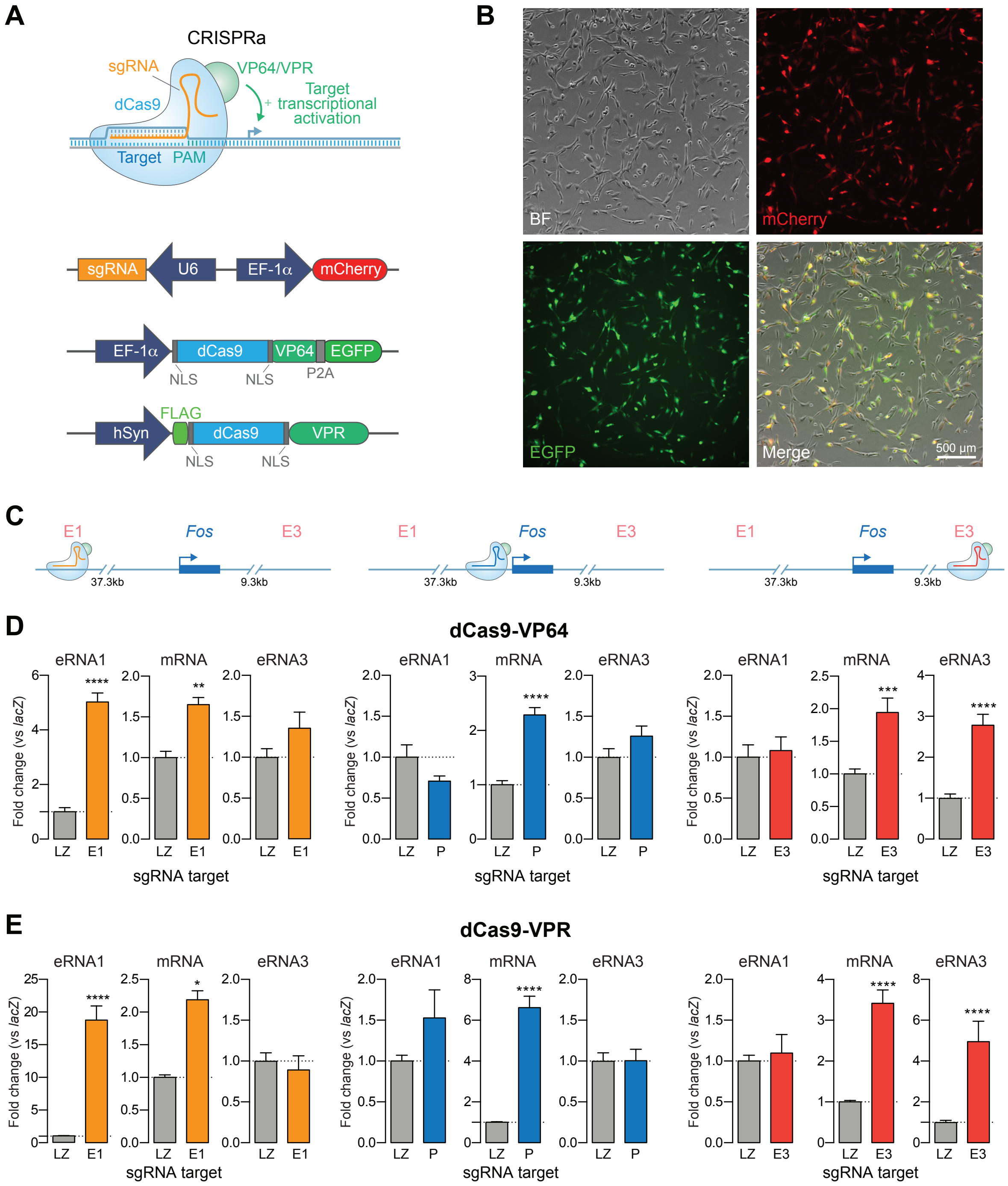

Supplementary Figure 3. Enhancer activation increases *Fos* eRNA and mRNA expression. **A**, Illustration of CRISPR activation (CRISPRa) strategy for site-specific targeting of the transcriptional activator VP64 or VPR. **B**, C6 cells 16 hrs post nucleofection with VP64 containing plasmids (dCas-VP64 expression marked by GFP reporter, gRNA expression marked by mCherry reporter). **C**, sgRNA locations for *Fos* enhancer and promoter targeting. **D**, RT-qPCR analysis of VP64 mediated induction of *Fos* eRNAs and mRNA when targeted to individual sites surrounding the *Fos* gene, compared to the non-targeting *lacZ* control. CRISPRa resulted in site-specific upregulation of selected eRNAs and mRNA. Increasing *Fos* eRNA1 and eRNA3 levels resulted in increased *Fos* mRNA levels but not vice versa (n = 9 per group; one-way ANOVA for eRNA1 ($F(4,40) = 66.22$, $p < 0.0001$), eRNA3 ($F(4,40) = 10.55$, $p < 0.0001$), and mRNA ($F(4,40) = 14.66$, $p < 0.0001$); Dunnett's multiple comparisons test). **E**, RT-qPCR analysis of VPR mediated induction of *Fos* eRNAs and mRNA when targeted to individual sites. CRISPRa resulted in site specific upregulation of selected eRNAs and mRNA compared to non-targeting *lacZ* control (n = 9 per group; one-way ANOVA for eRNA1 ($F(4,40) = 49.47$, $p < 0.0001$), eRNA3 ($F(4,40) = 18.52$, $p < 0.0001$), and mRNA ($F(4,40) = 46.43$, $p < 0.0001$); Dunnett's multiple comparisons test. Increasing *Fos* eRNA1 and eRNA3 levels resulted in increased mRNA levels but not vice versa. Data expressed as mean ± s.e.m. Multiple comparisons, *$p < 0.05$, **$p < 0.01$, ***$p < 0.001$, ****$p < 0.0001$.

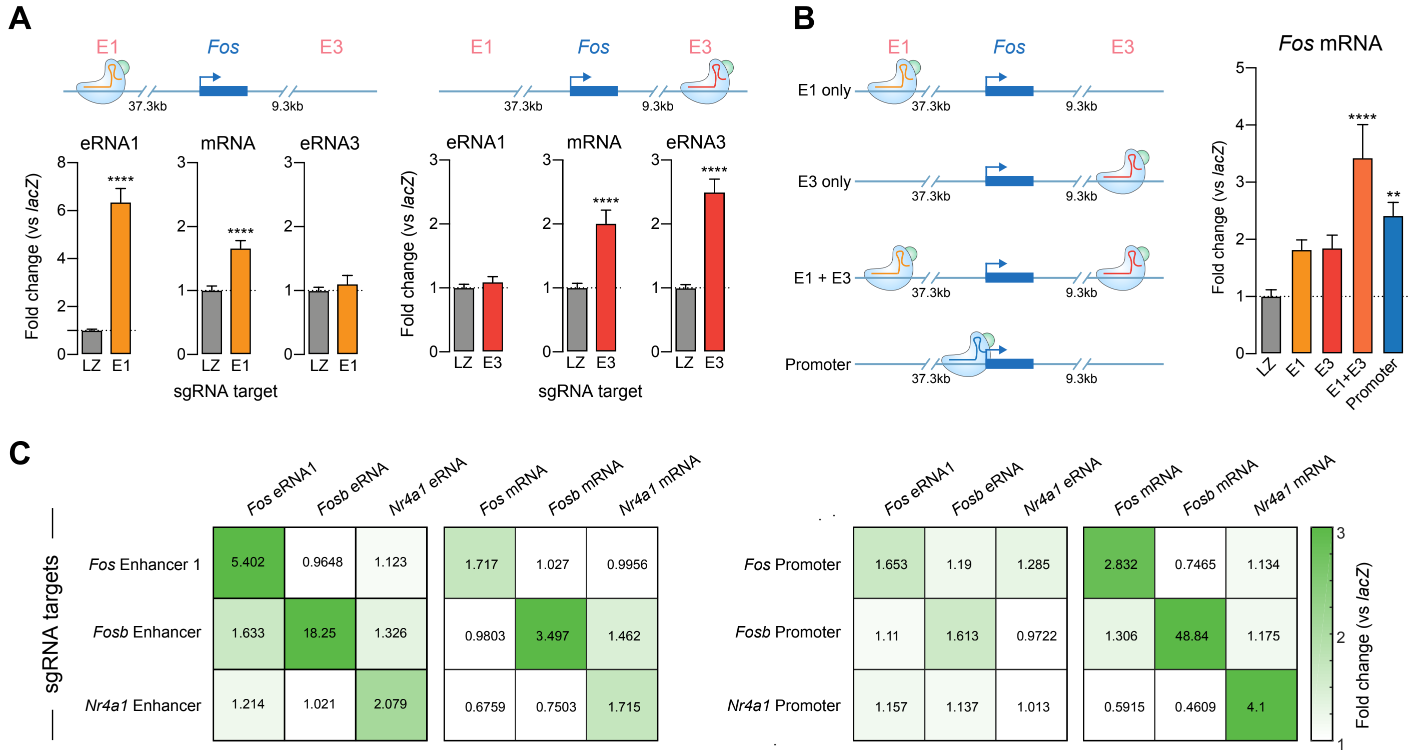

Supplementary Figure 4. CRISPRa selectively activates targeted enhancer and linked gene without altering other enhancers or genes. **A**, CRISPRa targeting at a distal upstream enhancer (left) or a downstream enhancer (right) at the *Fos* gene locus. VPR targeting to enhancers induced robust eRNA transcription and also increased mRNA levels. Notably, *Fos* E1 targeting did not induce *Fos* eRNA3, and vice versa. Gene expression differences were measured with RT-qPCR (n = 18 per group; two-tailed Mann-Whitney test for all comparisons, *Fos* E1-eRNA3 U = 161, p = 0.9875; *Fos* P-eRNA1 U = 143, p = 0.5628; *Fos* P-eRNA3 U = 116, p = 0.1516; some data repeated from Fig.4). **B**, Multiplexed VPR-mediated enhancer activation in primary cortical neurons resulted in additive increases in *Fos* mRNA (n = 9 per group, Kurskal-Wallis $F(4,40)$ = 25.04, p < 0.0001; Dunn's multiple comparisons test). **C**, RT-qPCR data heatmap of CRISPRa experiments demonstrating specificity of enhancer (left) and promoter (right) activation. Enhancer activation induced eRNAs and mRNAs at the target genes with little effect on other tested eRNA or mRNAs. Promoter activation produced increases in mRNA with little effect on eRNA levels. Data expressed as mean ± s.e.m. Multiple comparisons, *p < 0.05, **p < 0.01, ***p < 0.001, ****p

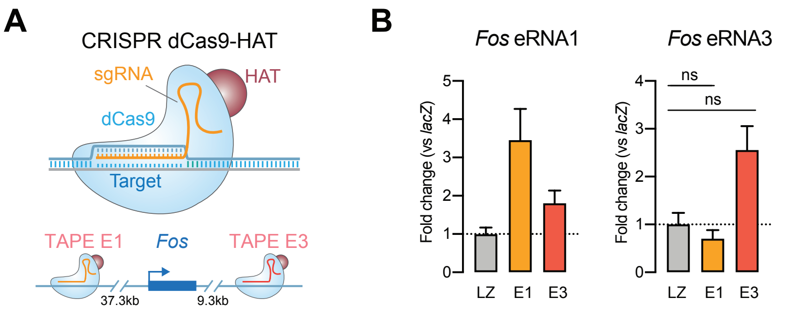

Supplementary Figure 5. eRNA levels in response to HAT recruitment to enhancers. **A**, Illustration of CRISPR dCas-HAT targeting in C6 cells, in which dCas9 carrying a histone acetyltransferase domain is targeted to *Fos* enhancer 1 and 3. **B**, HAT recruitment yielded no significant changes in eRNA1 (left, n = 8-9 per group, Kruskal-Wallis $F(2,23) = 4.041$ $p = 0.1326$) and eRNA3 (right, n = 8-9 per group, Kruskal-Wallis $F(2,23) = 7.87$ $p = 0.0195$, no significance in multiple comparisons). Data expressed as mean ± s.e.m. Multiple comparisons, *$p < 0.05$, **$p < 0.01$, ***$p < 0.001$, ****$p < 0.0001$.

Supplementary methods

Enhancer RNAs predict enhancer-gene regulatory links and are critical for enhancer function in neuronal systems

Nancy V.N. Carullo[1], Robert A. Phillips III[1], Rhiana C. Simon[1], Salomon Roman[1], Jenna Hinds[1], Aaron J. Salisbury[1], Jasmin S. Revanna[1], Kendra D. Bunner[1], Lara Ianov[2], Faraz A. Sultan[1], Katherine E. Savell[1], Charles A. Gersbach[3], & Jeremy J. Day[1,2]*

Enhancer Identification Pipeline

This analysis pipeline uses both ATAC-seq and total RNA-seq sequencing libraries from rat primary cortex, hippocampus, and striatum (removed at E18, cultured for 11 DIV prior to 1hr treatment with either vehicle or 10mM potassium chloride).

Step 1. Use SAMtools to merge ATAC-seq BAM files into single file for each primary neuron culture system.

- Cortex = Cor, 6 files (3 vehicle, 3 KCl)
- Hippocampus = Hpc, 6 files (3 vehicle, 3 KCl)
- Striatum = Str, 6 files (3 vehicle, 3 KCl)

```
module load SAMtools

samtools merge Cor_ATAC_merged.bam  ATAC_K2_S1.bam ATAC_K3_S2.bam
ATAC_K4_S3.bam ATAC_V1_S4.bam ATAC_V2_S5.bam ATAC_V3_S6.bam

samtools sort Cor_ATAC_merged.bam -o Cor_ATAC_merged.sorted.bam

samtools index Cor_ATAC_merged.sorted.bam
Cor_ATAC_merged.sorted.bam.bai

samtools merge Hpc_ATAC_merged.bam AS0011_K1_S2.bam
AS0011_K2_S4.bam AS0011_K4_S6.bam AS0011_V1_S1.bam
AS0011_V2_S3.bam AS0011_V4_S5.bam

samtools sort Hpc_ATAC_merged.bam -o Hpc_ATAC_merged.sorted.bam

samtools index Hpc_ATAC_merged.sorted.bam
Hpc_ATAC_merged.sorted.bam.bai

samtools merge Str_ATAC_merged.bam AS0008_K1_S11.bam
AS0008_K2_S12.bam AS0008_K3_S13.bam AS0008_V1_S14.bam
AS0008_V2_S15.bam AS0008_V4_S16.bam

samtools sort Str_ATAC_merged.bam -o Str_ATAC_merged.sorted.bam

samtools index Str_ATAC_merged.sorted.bam
Str_ATAC_merged.sorted.bam.bai
```

Step 2. Call ATAC-seq peaks with MACS2 using merged files from each primary neuron culture system.

```
module load MACS2

macs2 callpeak \
        --treatment
/data/user/rphill3/Enhancer_Ident_ATAC/Str_ATAC_merged.bam \
        --qvalue 0.00001 \
        --gsize 2729862089 \
        --format BAMPE \
        --outdir
/data/user/rphill3/Enhancer_Ident_ATAC/Final_PeakCalls \
        --name Str_Merged_BAMPE_narrow_0.00001_FinalPeaks

macs2 callpeak \
        --treatment
/data/user/rphill3/Enhancer_Ident_ATAC/Cor_ATAC_merged.bam \
        --qvalue 0.00001 \
        --gsize 2729862089 \
        --format BAMPE \
        --outdir
/data/user/rphill3/Enhancer_Ident_ATAC/Final_PeakCalls \
        --name Cor_Merged_BAMPE_narrow_0.00001_FinalPeaks


macs2 callpeak \
        --treatment
/data/user/rphill3/Enhancer_Ident_ATAC/Hpc_ATAC_merged.bam \
        --qvalue 0.00001 \
        --gsize 2729862089 \
        --format BAMPE \
        --outdir
/data/user/rphill3/Enhancer_Ident_ATAC/Final_PeakCalls \
        --name Hpc_Merged_BAMPE_narrow_0.00001_FinalPeaks
```

Step 3. Using MACS2 ATAC-seq peaks identified in each primary neuron culture system, merge peaks within 1kb using BEDtools.

```
#Load bedtools
module load BEDTools

cd /data/user/rphill3/Enhancer_Ident_ATAC/Final_PeakCalls

for i in *.narrowPeak.bed
do
#sort the bed
sort -k1,1 -k2,2n $i > Sorted_beds/$i.sorted.bed
#merge peaks within 1000bps of each other
bedtools merge -i Sorted_beds/$i.sorted.bed -d 1000 >
Merged_beds/$i.sorted.merged.bed
done
```

Steps 4-5. Using base R script, remove ATAC-seq peaks smaller than 146bp (length of DNA wrapped around nucleosome). Next, use rbind to combine ATAC-seq MACS2 peaks from all regions into one file. These are termed "Regions of Open Chromatin (ROCs)".

```
#BAM files for individual samples were merged using samtools.
Then, MACS2 was used to callpeaks. Peaks <=1000bps apart were
merged within region. Here, I will read in the merged peak files,
create a length column, and
#subset peaks that are >146bps, or the length of the DNA wrapped
around a single nucleosome.
#Str
Str_Merged_1Kb_Peaks <- read.delim(file =
"/Volumes/JDLab$/RobertPhillips/Bioinformatics/JD0032_EnhancerIde
ntification/PeakCalls_Merged_WithinRegion/Str_Merged_BAMPE_narrow
_0.00001_FinalPeaks_peaks.narrowPeak.bed.sorted.merged.bed",heade
r = FALSE)
Str_Merged_1Kb_Peaks$Length <- Str_Merged_1Kb_Peaks$V3 -
Str_Merged_1Kb_Peaks$V2
Str_Merged_1Kb_Peaks <- subset(Str_Merged_1Kb_Peaks,subset =
(Length >= 146))
write.table(x = Str_Merged_1Kb_Peaks,
            file =
"/Volumes/JDLab$/RobertPhillips/Bioinformatics/JD0032_EnhancerIde
ntification/PeakCalls_Merged_WithinRegion/SizeSelected/Str_Merged
_BAMPE_narrow_0.00001_FinalPeaks_peaks.narrowPeak.bed.sorted.merg
ed.sizeselected.bed",
            quote = FALSE,
            row.names = FALSE,
            col.names = FALSE,
            sep = "\t")
#Cor
Cor_Merged_1Kb_Peaks <- read.delim(file =
"/Volumes/JDLab$/RobertPhillips/Bioinformatics/JD0032_EnhancerIde
ntification/PeakCalls_Merged_WithinRegion/Cor_Merged_BAMPE_narrow
_0.00001_FinalPeaks_peaks.narrowPeak.bed.sorted.merged.bed",heade
r = FALSE)
Cor_Merged_1Kb_Peaks$Length <- Cor_Merged_1Kb_Peaks$V3 -
Cor_Merged_1Kb_Peaks$V2
Cor_Merged_1Kb_Peaks <- subset(Cor_Merged_1Kb_Peaks,subset =
(Length >= 146))
write.table(x = Cor_Merged_1Kb_Peaks,
            file =
"/Volumes/JDLab$/RobertPhillips/Bioinformatics/JD0032_EnhancerIde
ntification/PeakCalls_Merged_WithinRegion/SizeSelected/Cor_Merged
_BAMPE_narrow_0.00001_FinalPeaks_peaks.narrowPeak.bed.sorted.merg
ed.sizeselected.bed",
            quote = FALSE,
            row.names = FALSE,
            col.names = FALSE,
            sep = "\t")
#Hpc
Hpc_merged_1Kb_Peaks <- read.delim(file =
"/Volumes/JDLab$/RobertPhillips/Bioinformatics/JD0032_EnhancerIde
ntification/PeakCalls_Merged_WithinRegion/Hpc_Merged_BAMPE_narrow
```

```
_0.00001_FinalPeaks_peaks.narrowPeak.bed.sorted.merged.bed",heade
r = FALSE)
Hpc_merged_1Kb_Peaks$Length <- Hpc_merged_1Kb_Peaks$V3 -
Hpc_merged_1Kb_Peaks$V2
Hpc_merged_1Kb_Peaks <- subset(Hpc_merged_1Kb_Peaks,subset =
(Length >= 146))
write.table(x = Hpc_merged_1Kb_Peaks,
            file =
"/Volumes/JDLab$/RobertPhillips/Bioinformatics/JD0032_EnhancerIde
ntification/PeakCalls_Merged_WithinRegion/SizeSelected/Hpc_Merged
_BAMPE_narrow_0.00001_FinalPeaks_peaks.narrowPeak.bed.sorted.merg
ed.sizeselected.bed",
            quote = FALSE,
            row.names = FALSE,
            col.names = FALSE,
            sep = "\t")

#Now rbind all Peaks
All_Regions <-
rbind(Str_Merged_1Kb_Peaks,Cor_Merged_1Kb_Peaks,Hpc_merged_1Kb_Pe
aks)
write.table(x = All_Regions,
            file =
"/Volumes/JDLab$/RobertPhillips/Bioinformatics/JD0032_EnhancerIde
ntification/PeakCalls_Merged_WithinRegion/SizeSelected/AllRegions
_BAMPE_narrow_0.00001_FinalPeaks_peaks.narrowPeak.bed.sorted.merg
ed.sizeselected.bed",
            quote = FALSE,
            row.names = FALSE,
            col.names = FALSE,
            sep = "\t")
```

Step 6. Using Seqmonk, import all total RNA-seq BAM files as RNA-seq data. Create a merged file for all primary culture systems (Cortex, Hippocampus, Striatum) and treatment groups (vehicle or KCl).

- Cortex, 6 files (3 vehicle, 3 KCl)
- Hippocampus, 6 files (3 vehicle, 3 KCl)
- Striatum, 7 files (3 vehicle, 4 KCl)
- Import the following annotation files:
    - MACS2 ATAC-seq peaks from **Step 5** - AllRegions_ATACPeaks.bed
    - RefSeq curated gene annotations - RefSeq_Curated.bed
    - UCSC gene annotations - UCSC_RefSeq.bed
    - Ensemble gene annotations - Rn6_v95_gtf_genes.txt
    - Contiguously transcribed regions over 1kb – Contigs.txt

1. Import All BAM files
    - Do not remove duplicate reads
    - Do not treat as HiC data
    - Min mapping quality - 20
    - Import primary alignments only

- Treat as RNA-Seq data
- Data Type – Paired end
- Pair Distance Cutoff (bp) – n/a

2. Merge all files to make a single group track consisting of Veh and KCl samples from each regions using: Edit → Groups
3. Make probes around regions of open chromatin (ROCs)
   i. Data → Define Probes → Feature Probe Generator
   ii. Features to design around → AllRegions_ATACPeaks.bed
   iii. Split into subfeatures → No
   iv. Remove Exact duplicates → Yes
   v. Ignore feature strand information → No
   vi. Make Probes → Over Feature +/-500 bp
   vii. Identifies **191,857** probes → Name "ROCs"
   viii. Add ROCs to annotation track (Right click probe list, select "Convert to annotation track")
4. Filter ROCs that were are 1kbp of Refseq curated genes (Filtering → Filter by features)
   i. Features to design around – RefSeq_Curated.bed
   ii. Split into subfeatures – No
   iii. Make Probes- Over feature +/-1000bp
   iv. Select probes which are – overlapping
   v. Distance cutoff (bp) – n/a
   vi. Use features on strand – Any
   vii. Identifies 68,856 ROCs that are overlapping or within 1kb of RefSeq genes
5. Filter ROCs that were within 1kbp of UCSC genes (Filtering → Filter by features)
   i. Features to design around – UCSC_RefSeq.bed
   ii. Split into subfeatures – No
   iii. Make Probes- Over feature +/-1000bp
   iv. Select probes which are – overlapping
   v. Distance cutoff (bp) – n/a
   vi. Use features on strand – Any
   vii. Identifies 71,461 ROCs that are overlapping or within 1kb of UCSC genes
6. Filter ROCs that within 1kbp of Ensemble genes (Filtering → Filter by features)
   i. Features to design around – Rn6_v95_gtf_genes.txt
   ii. Split into subfeatures – No
   iii. Make Probes- Over feature +/-1000bp
   iv. Select probes which are – Overlapping
   v. Distance cutoff (bp) – n/a
   vi. Use features on strand – Any
   vii. Identifies 76,382 ROCs overlapping or within 1kb of Ensemble genes
7. Filtered ROCs that overlap Contiguously transcribed regions (Filtering → Filter by features)
   i. Features to design around – Contigs.txt
   ii. Split into subfeatures – No
   iii. Make Probes- Over feature +/-1000bp
   iv. Select probes which are – Overlapping
   v. Distance cutoff (bp) – n/a
   vi. Use features on strand – Any

    vii. Identifies 57366 ROCs overlapping or within 1kb of Contiguously transcribed regions
8. Repeat filtering for miRNA, misc_RNA, rRNA, snoRNA, snRNA, tRNA (Filtering → Filter by features)
    i. Features to design around – pick from above annotations provided by Seqmonk Rn6 genome assembly
    ii. Split into subfeatures – No
    iii. Make Probes- Over feature From +/- 0bp
    iv. Select probes which are – Overlapping
    v. Distance cutoff (bp) – n/a
    vi. Use features on strand – Any
    vii. 839 ROCs overlapping various ncRNA genes
9. Filtering → intersect multiple lists…
    i. Include ROCs, exclude all ROCs overlapping features from sections 4-8 of Step 6
    ii. Intersection leaves **100,767** "Intergenic ROCs" or iROCs
    iii. Convert to annotation track (Right click probe list, select "Convert to annotation track")

Steps 7-8. Using Seqmonk, quantify total RNA-seq transcription from iROCs. Then, filter for iROCs that are bidirectionally transcribed.

1. Design probes around iROCs (Data → Define Probes → Feature Probe Generator)
    i. Features to design around → iROCs
    ii. Split into subfeatures → No
    iii. Remove Exact duplicates → Checked
    iv. Ignore feature strand information → Unchecked
    v. Make Probes → Over Feature from -0 to +0 bp
2. Quantify all sequenced transcripts (Probe quantitation → Difference Quantitation)
    i. Calculate Forward only as a percentage of All reads
    ii. Min count = 0
    iii. Ignore duplicates – unchecked
3. Filter unidirectionally transcribed iROCs (Filtering → Filter on Values)
    i. Merged RNA-seq group value must be between 5 and 95%
    ii. This excludes probes with unidirectional transcription from either strand (likely unannotated genes or exons)
    iii. Identifies **28,492** "Transcriptionally active putative enhancers" – TAPEs
    iv. Convert to annotation track Right click probe list, select "Convert to annotation track"
4. Make probes defining TAPEs (Define probes → Feature Probe Generator)
    i. Features to design around → "TAPEs"
    ii. Split into subfeatures → No
    iii. Remove exact duplicates → Checked
    iv. Ignore strand feature information → Unchecked
    v. Make probes → Over feature -0 to +0 bp
5. Quantify TAPE transcription (CPKM) (Data → Read count quantitation)
    i. Count reads on strand → All reads
    ii. Correct for total read count → Checked

iii. Correct to what? → per million reads
iv. Count total only in probes? → Unchecked
v. Correct for probe length? → Checked
vi. Log transform count → Unchecked
vii. Count duplicate reads only once → Unchecked
6. Filtering → Probe Values filter
   a. RNA-seq group value must be between 0.05 and 10000
   b. Identifies 1916 highly expressed TAPEs
7. Create report for TAPEs
   c. Reports → Annotated Probe Report
   d. Annotate with overlapping Rn6_v95_gtf_genes.txt
   e. Annotation distance cutoff – 1Mbp
   f. Include – unannotated probes
   g. Include – data for currently visible stores

Steps 9. Using R, identify all genes +/- 1Mbp of TAPEs. Then, correlate transcription at each TAPE with transcription at each gene.

Mapping Transcriptionally Active Putative Enhancers (TAPEs) to Genes

The goal of this analysis is to identify high confidence TAPE-gene pairs. To do this, transcription start sites located within 1Mb upstream or downstream from the center of the TAPE are identified. Then, pearson's correlations are calculated using counts for the TAPEs and associated genes

Load Libraries

First, all essential libraries are loaded for the analysis.

```
suppressPackageStartupMessages(library(dplyr))
suppressPackageStartupMessages(library(Seurat))

## Warning: package 'Seurat' was built under R version 3.6.2

suppressPackageStartupMessages(library(ggplot2))
suppressPackageStartupMessages(library(cowplot))
suppressPackageStartupMessages(library(data.table))
suppressPackageStartupMessages(library(VennDiagram))
```

Load in Required Data

First, read in the 28,492 TAPEs identified in the **Step 8.**

```
TAPEs <- read.table(file = "~/TAPE_RNA_quantification_CPKM.txt",sep = "\t",he
ader = TRUE)
```

Now we are going to create a column indicating these as intergenic TAPEs. Then, we are going to create an ID column consisting of the TAPE chromosome, starting position, and ending position. Next, we will rename the first four columnns. The start and end will be referred to as five and three prime end as the SeqMonk software used during the pipeline refers to the 5' end of all genes as the start, regardless of whether that gene is on the - strand. Thus, referring to the start/end as five prime

and three prime will allow us to correctly calculate distance later in this workflow. Finally, the center of the TAPE is calculated.

```
#Give a column indicating the type
TAPEs$Type <- "Intergenic"

#Create a column with a unique ID.
TAPEs$ID <- paste(TAPEs$Chromosome,TAPEs$Start,TAPEs$End,sep = "_")

#Change the TAPEs names
names(TAPEs)[1:4] <- c("TAPE_ID","TAPE_Chr","TAPE_Five_Prime_End","TAPE_Three
_Prime_End")

#Calculate the center of the TAPE
TAPEs$TAPE_Center <- TAPEs[,"TAPE_Five_Prime_End"] + (round(abs(TAPEs[,"TAPE_
Five_Prime_End"] - TAPEs[,"TAPE_Three_Prime_End"])/2))
```

Next, read in the genes used during TAPE identification and change the column names.

```
#read in the genes that were used to during enhancer identificaiton
genes <- read.delim(file = "~/Rn6_v95_gtf_genesonly_noBS.txt",sep = "\t")

#Change column naems
names(genes) <- c("Chr","Gene_Five_Prime_End","Gene_Three_Prime_End","Strand"
,"Gene")
```

To map the TAPEs to associated genes, we will use a for loop. As the for loop proceeds, any TAPE that maps to the gene will be input into a large list created below. Here, every element of the list corresponds to a unique gene.

```
# Here I make an empty list to input the annotated TAEs into
genes_list <- vector(mode   = "list",
                     length = nrow(genes))
#Name every element of the list a unique name for that gene. This consists of
chr,5' end, 3' end, Gene name, and strand that is comma separated
names(genes_list) <- paste(paste(paste(paste(genes$Chr,genes$Gene_Five_Prime_
End,sep = ","),
                                       genes$Gene_Three_Prime_End,sep = ","),
                                 genes$Gene,sep = ","),
                           genes$Strand,sep = ",")
```

Next we run a sanity check to make sure that the dataframe and list are in the same order.

```
#Make sure the dataframe and list are in the same order
all(as.character(lapply(strsplit(names(genes_list),split = ","),"[",4)) == ge
nes$Gene) # TRUE

## [1] TRUE

all(as.character(lapply(strsplit(names(genes_list),split = ","),"[",5)) == ge
nes$Strand) # TRUE

## [1] TRUE

all(as.character(lapply(strsplit(names(genes_list),split = ","),"[",1)) == ge
nes$Chr) # TRUE
```

```
## [1] TRUE

all(as.numeric(lapply(strsplit(names(genes_list),split = ","),"[",2))    == ge
nes$Gene_Five_Prime_End) # TRUE

## [1] TRUE

all(as.numeric(lapply(strsplit(names(genes_list),split = ","),"[",3))    == ge
nes$Gene_Three_Prime_End) # TRUE

## [1] TRUE
```

Identify TAPE-Gene Pairs

Now we annotate all genes that are 1Mbp upstream and downstream of the TAPE. This loops through the genes dataframe, and first asks if the strand of the gene is + or -. If the strand of the gene is positive all calculations are computed using the five prime end of the gene. If the strand of the gene is negative all calculations are computed using the three prime end of the gene. This search is gene-centric in that this loop identifies genes that fall within 1Mbp windows from the center of the TAPE.A progress bar will also print the progress of the loop.

Next, we identify any genes in which there were no associated TAPEs. These empty elements are then removed from the list.

```
#Empty vector for identification of empty list elements
x <-vector()
#Run loop
for(i in 1:length(genes_list)){
  if(is.null(genes_list[[i]])){
    x <- append(x = x,i)
  }else{
    next
  }
}
#Remove genes with no TAPEs
genes_list <- genes_list[-x]
```

This for loop adds a column to every dataframe in the list that indicates the gene name, strand, chr, 5'end, 3' end. The list is then unlisted to create a large dataframe that can be exported. Finally, a sanity check is run to make sure that no rows are duplicated.

```
for(i in 1:length(genes_list)){
  genes_list[[i]]$Gene                 <- as.character(lapply(strsplit(names(
genes_list)[i],split = ","),"[",4))
  genes_list[[i]]$Strand               <- as.character(lapply(strsplit(names(
genes_list)[i],split = ","),"[",5))
  genes_list[[i]]$Gene_Chr             <- as.character(lapply(strsplit(names(
genes_list)[i],split = ","),"[",1))
  genes_list[[i]]$Gene_Five_Prime_End  <- as.numeric(lapply(strsplit(names(ge
nes_list)[i],split = ","),"[",2))
  genes_list[[i]]$Gene_Three_Prime_End <- as.numeric(lapply(strsplit(names(ge
nes_list)[i],split = ","),"[",3))
}
```

```
#unlist and make a huge dataframe
TAPEs_df <- rbindlist(genes_list)

#There should not be any duplicated rows, but this command is a sanity check
TAPEs_df <- as.data.frame(distinct(TAPEs_df)) #433,416
```

Here distance is calculated. If the gene is on the + strand, the TSS is the five prime end of the gene. If the gene is on the - strand, the TSS is the three prime end of the gene.

```
# Calculate distance and orientation based on strand
TAPEs_df$Distance    <- NA
TAPEs_df$Orientation <- NA
TAPEs_df$Orientation <- as.character(TAPEs_df$Orientation)
#Calculate distance and orientation based on strand
#To calculate the distance, the TSS for each gene must be identified. The TSS
changes for each gene's strandedness in that a + stand gene's TSS will be in
the Gene_Five_Prime_End column and a - strand gene's TSS will be in the Gene_
Three_Prime_End column
TAPEs_df$TSS <- NA
TAPEs_df <- TAPEs_df %>% mutate(TSS = ifelse(Strand == "+",
                                              TAPEs_df$Gene_Five_Prime_End,
                                              TAPEs_df$Gene_Three_Prime_End))
```

Next, the orientation of the gene to the TAPE is identified and distance from center of TAPE to TSS of gene is calculated.

```
# #If the Gene's TSS is < the TAPE center and > TAPE-1e6 then the gene is ups
tream of the enhancer
# #If the Gene's TSS is > the TAPE center and < TAPE+1e6 then the gene is dow
nstream of the enhancer
TAPEs_df <- TAPEs_df %>% mutate(Orientation = ifelse((TAPEs_df$TSS < TAPEs_df
$TAPE_Center) & (TAPEs_df$TSS > (TAPEs_df$TAPE_Center - 1e+06)),
                                                     "Upstream",
                                                     ifelse((TAPEs_df$TSS > T
APEs_df$TAPE_Center) & (TAPEs_df$TSS < (TAPEs_df$TAPE_Center+1e+06)),
                                                            "Downstream",
                                                            0)
)
)
#Calculate Distance
TAPEs_df <- TAPEs_df %>% mutate(Distance = ifelse(Orientation == "Upstream",
                                                  TAPEs_df$TAPE_Center - TAPE
s_df$TSS,
                                                  TAPEs_df$TSS - TAPEs_df$TAP
E_Center))
#Another sanity check that the chromosomes of the TAPEs and the genes are the
same
table(as.character(TAPEs_df$TAPE_Chr) == as.character(TAPEs_df$Gene_Chr))

##
##   TRUE
## 433416
```

To calculate correlations we need TAPE and gene counts. The TAPE counts are already within the TAPEs dataframe. Here, we load in mRNA count information. Next, we keep only genes in which there is one annotation. Finally, only useful columns are kept.

```r
#Read in the Gene Probe counts identified with Seqmonk
Gene_Probe_Counts <- read.delim(file = "~/mRNA_quantification_CPKM.txt",sep =
"\t")
#Keep only genes in which there is one annotated gene.
Gene_Probe_Counts <- Gene_Probe_Counts[!duplicated(as.character(Gene_Probe_Co
unts$Probe)),]
#Pull out useful columns Probe, Sample Counts
Gene_Probe_Counts <- Gene_Probe_Counts[,c(1,13:31)]
```

Within this loop, Pearson's correlations are calculated.

```r
#Make columns for correlations
TAPEs_df$Cortex_Correlation      <- NA
TAPEs_df$Hippocampus_Correlation <- NA
TAPEs_df$Striatum_Correlation    <- NA
TAPEs_df$Global_Correlation      <- NA
x <- vector()
#Now calculate correlations
for(i in 1:nrow(TAPEs_df)){
  #figure out which row in the Gene_Probe_Counts column corresponds to the ge
ne in the TAPEs_df column
  row <- which(as.character(TAPEs_df[i,"Gene"]) == as.character(Gene_Probe_Co
unts$Probe))
  if(length(row) >0){
    #####Cortex####
    #Calculate the correlation
    TAPEs_df[i,"Cortex_Correlation"] <- cor(y = as.numeric(TAPEs_df[i,c(13:18
)]),x = as.numeric(Gene_Probe_Counts[row,c(2:7)]))
    #####Hippocampus######
    #Calculate the correlation
    TAPEs_df[i,"Hippocampus_Correlation"] <- cor(y = as.numeric(TAPEs_df[i,c(
19:24)]),x = as.numeric(Gene_Probe_Counts[row,c(8:13)]))
    #######Striatum#######
    #Calculate the correlation
    TAPEs_df[i,"Striatum_Correlation"]  <- cor(y = as.numeric(TAPEs_df[i,c(25
:31)]),x = as.numeric(Gene_Probe_Counts[row,c(14:20)]))
    #Gloabl Correlation
    #calculate the correlation
    TAPEs_df[i,"Global_Correlation"] <- cor(y = as.numeric(TAPEs_df[i,13:31])
,x = as.numeric(Gene_Probe_Counts[row,2:20]))
  }else{
    x <- append(x = x,i)
  }
}
```

Some of the genes have count values of 0 for every sample across all cell types. This results in a correaltion value of 0. Thus, these TAPE-gene pairs are removed, leaving us with 388,605 potential TAPE-gene pairs.

```r
TAPEs_df <- TAPEs_df[!is.na(TAPEs_df$Global_Correlation),]
nrow(TAPEs_df) #388,605

## [1] 388605
```
