# Supplementary material for: Enhancer RNAs predict enhancer–gene regulatory links and are critical for enhancer function in neuronal systems
Source: Nucleic Acids Res. 2020 Aug 18;48(17):9550–70. doi: 10.1093/nar/gkaa671 (PMC7515708; doi:10.1093/nar/gkaa671)
Supplement: gkaa671_Supplemental_Files [file gkaa671_supplemental_files.zip › Supplementary Methods.docx]

**Supplementary methods**

**Enhancer RNAs predict enhancer-gene regulatory links and are critical for enhancer function in neuronal systems**

Nancy V.N. Carullo^1^, Robert A. Phillips III^1^, Rhiana C. Simon^1^, Salomon Roman^1^, Jenna Hinds^1^, Aaron J. Salisbury^1^, Jasmin S. Revanna^1^, Kendra D. Bunner^1^, Lara Ianov^2^, Faraz A. Sultan^1^, Katherine E. Savell^1^, Charles A. Gersbach^3^, & Jeremy J. Day^1,2^*

**Enhancer Identification Pipeline**

This analysis pipeline uses both ATAC-seq and total RNA-seq sequencing libraries from rat primary cortex, hippocampus, and striatum (removed at E18, cultured for 11 DIV prior to 1hr treatment with either vehicle or 10mM potassium chloride).

**Step 1.** Use SAMtools to merge ATAC-seq BAM files into single file for each primary neuron culture system.

- Cortex = Cor, 6 files (3 vehicle, 3 KCl)
- Hippocampus = Hpc, 6 files (3 vehicle, 3 KCl)
- Striatum = Str, 6 files (3 vehicle, 3 KCl)

module load SAMtools

samtools merge Cor_ATAC_merged.bam ATAC_K2_S1.bam ATAC_K3_S2.bam ATAC_K4_S3.bam ATAC_V1_S4.bam ATAC_V2_S5.bam ATAC_V3_S6.bam

samtools sort Cor_ATAC_merged.bam -o Cor_ATAC_merged.sorted.bam

samtools index Cor_ATAC_merged.sorted.bam Cor_ATAC_merged.sorted.bam.bai

samtools merge Hpc_ATAC_merged.bam AS0011_K1_S2.bam AS0011_K2_S4.bam AS0011_K4_S6.bam AS0011_V1_S1.bam AS0011_V2_S3.bam AS0011_V4_S5.bam

samtools sort Hpc_ATAC_merged.bam -o Hpc_ATAC_merged.sorted.bam

samtools index Hpc_ATAC_merged.sorted.bam Hpc_ATAC_merged.sorted.bam.bai

samtools merge Str_ATAC_merged.bam AS0008_K1_S11.bam AS0008_K2_S12.bam AS0008_K3_S13.bam AS0008_V1_S14.bam AS0008_V2_S15.bam AS0008_V4_S16.bam

samtools sort Str_ATAC_merged.bam -o Str_ATAC_merged.sorted.bam

samtools index Str_ATAC_merged.sorted.bam Str_ATAC_merged.sorted.bam.bai

**Step 2.** Call ATAC-seq peaks with MACS2 using merged files from each primary neuron culture system.

module load MACS2

macs2 callpeak \

--treatment /data/user/rphill3/Enhancer_Ident_ATAC/Str_ATAC_merged.bam \

--qvalue 0.00001 \

--gsize 2729862089 \

--format BAMPE \

--outdir /data/user/rphill3/Enhancer_Ident_ATAC/Final_PeakCalls \

--name Str_Merged_BAMPE_narrow_0.00001_FinalPeaks

macs2 callpeak \

--treatment /data/user/rphill3/Enhancer_Ident_ATAC/Cor_ATAC_merged.bam \

--qvalue 0.00001 \

--gsize 2729862089 \

--format BAMPE \

--outdir /data/user/rphill3/Enhancer_Ident_ATAC/Final_PeakCalls \

--name Cor_Merged_BAMPE_narrow_0.00001_FinalPeaks

macs2 callpeak \

--treatment /data/user/rphill3/Enhancer_Ident_ATAC/Hpc_ATAC_merged.bam \

--qvalue 0.00001 \

--gsize 2729862089 \

--format BAMPE \

--outdir /data/user/rphill3/Enhancer_Ident_ATAC/Final_PeakCalls \

--name Hpc_Merged_BAMPE_narrow_0.00001_FinalPeaks

**Step 3.** Using MACS2 ATAC-seq peaks identified in each primary neuron culture system, merge peaks within 1kb using BEDtools.

#Load bedtools

module load BEDTools

cd /data/user/rphill3/Enhancer_Ident_ATAC/Final_PeakCalls

for i in *.narrowPeak.bed

do

#sort the bed

sort -k1,1 -k2,2n $i > Sorted_beds/$i.sorted.bed

#merge peaks within 1000bps of each other

bedtools merge -i Sorted_beds/$i.sorted.bed -d 1000 > Merged_beds/$i.sorted.merged.bed

done

**Steps 4-5.** Using base R script, remove ATAC-seq peaks smaller than 146bp (length of DNA wrapped around nucleosome). Next, use rbind to combine ATAC-seq MACS2 peaks from all regions into one file. These are termed “Regions of Open Chromatin (ROCs)”.

#BAM files for individual samples were merged using samtools. Then, MACS2 was used to callpeaks. Peaks <=1000bps apart were merged within region. Here, I will read in the merged peak files, create a length column, and

#subset peaks that are >146bps, or the length of the DNA wrapped around a single nucleosome.

#Str

Str_Merged_1Kb_Peaks <- read.delim(file = "/Volumes/JDLab$/RobertPhillips/Bioinformatics/JD0032_EnhancerIdentification/PeakCalls_Merged_WithinRegion/Str_Merged_BAMPE_narrow_0.00001_FinalPeaks_peaks.narrowPeak.bed.sorted.merged.bed",header = FALSE)

Str_Merged_1Kb_Peaks$Length <- Str_Merged_1Kb_Peaks$V3 - Str_Merged_1Kb_Peaks$V2

Str_Merged_1Kb_Peaks <- subset(Str_Merged_1Kb_Peaks,subset = (Length >= 146))

write.table(x = Str_Merged_1Kb_Peaks,

file = "/Volumes/JDLab$/RobertPhillips/Bioinformatics/JD0032_EnhancerIdentification/PeakCalls_Merged_WithinRegion/SizeSelected/Str_Merged_BAMPE_narrow_0.00001_FinalPeaks_peaks.narrowPeak.bed.sorted.merged.sizeselected.bed",

quote = FALSE,

row.names = FALSE,

col.names = FALSE,

sep = "\t")

#Cor

Cor_Merged_1Kb_Peaks <- read.delim(file = "/Volumes/JDLab$/RobertPhillips/Bioinformatics/JD0032_EnhancerIdentification/PeakCalls_Merged_WithinRegion/Cor_Merged_BAMPE_narrow_0.00001_FinalPeaks_peaks.narrowPeak.bed.sorted.merged.bed",header = FALSE)

Cor_Merged_1Kb_Peaks$Length <- Cor_Merged_1Kb_Peaks$V3 - Cor_Merged_1Kb_Peaks$V2

Cor_Merged_1Kb_Peaks <- subset(Cor_Merged_1Kb_Peaks,subset = (Length >= 146))

write.table(x = Cor_Merged_1Kb_Peaks,

file = "/Volumes/JDLab$/RobertPhillips/Bioinformatics/JD0032_EnhancerIdentification/PeakCalls_Merged_WithinRegion/SizeSelected/Cor_Merged_BAMPE_narrow_0.00001_FinalPeaks_peaks.narrowPeak.bed.sorted.merged.sizeselected.bed",

quote = FALSE,

row.names = FALSE,

col.names = FALSE,

sep = "\t")

#Hpc

Hpc_merged_1Kb_Peaks <- read.delim(file = "/Volumes/JDLab$/RobertPhillips/Bioinformatics/JD0032_EnhancerIdentification/PeakCalls_Merged_WithinRegion/Hpc_Merged_BAMPE_narrow_0.00001_FinalPeaks_peaks.narrowPeak.bed.sorted.merged.bed",header = FALSE)

Hpc_merged_1Kb_Peaks$Length <- Hpc_merged_1Kb_Peaks$V3 - Hpc_merged_1Kb_Peaks$V2

Hpc_merged_1Kb_Peaks <- subset(Hpc_merged_1Kb_Peaks,subset = (Length >= 146))

write.table(x = Hpc_merged_1Kb_Peaks,

file = "/Volumes/JDLab$/RobertPhillips/Bioinformatics/JD0032_EnhancerIdentification/PeakCalls_Merged_WithinRegion/SizeSelected/Hpc_Merged_BAMPE_narrow_0.00001_FinalPeaks_peaks.narrowPeak.bed.sorted.merged.sizeselected.bed",

quote = FALSE,

row.names = FALSE,

col.names = FALSE,

sep = "\t")

#Now rbind all Peaks

All_Regions <- rbind(Str_Merged_1Kb_Peaks,Cor_Merged_1Kb_Peaks,Hpc_merged_1Kb_Peaks)

write.table(x = All_Regions,

file = "/Volumes/JDLab$/RobertPhillips/Bioinformatics/JD0032_EnhancerIdentification/PeakCalls_Merged_WithinRegion/SizeSelected/AllRegions_BAMPE_narrow_0.00001_FinalPeaks_peaks.narrowPeak.bed.sorted.merged.sizeselected.bed",

quote = FALSE,

row.names = FALSE,

col.names = FALSE,

sep = "\t")

**Step 6.** Using Seqmonk, import all total RNA-seq BAM files as RNA-seq data. Create a merged file for all primary culture systems (Cortex, Hippocampus, Striatum) and treatment groups (vehicle or KCl).

- Cortex, 6 files (3 vehicle, 3 KCl)
- Hippocampus, 6 files (3 vehicle, 3 KCl)
- Striatum, 7 files (3 vehicle, 4 KCl)
- Import the following annotation files:
  - MACS2 ATAC-seq peaks from **Step 5** - AllRegions_ATACPeaks.bed
  - RefSeq curated gene annotations - RefSeq_Curated.bed
  - UCSC gene annotations - UCSC_RefSeq.bed
  - Ensemble gene annotations - Rn6_v95_gtf_genes.txt
  - Contiguously transcribed regions over 1kb – Contigs.txt

1. Import All BAM files
   - Do not remove duplicate reads
   - Do not treat as HiC data
   - Min mapping quality - 20
   - Import primary alignments only
   - Treat as RNA-Seq data
   - Data Type – Paired end
   - Pair Distance Cutoff (bp) – n/a
2. Merge all files to make a single group track consisting of Veh and KCl samples from each regions using: Edit 🡪 Groups
3. Make probes around regions of open chromatin (ROCs)
4. Data 🡪 Define Probes 🡪 Feature Probe Generator
5. Features to design around 🡪 AllRegions_ATACPeaks.bed
6. Split into subfeatures 🡪 No
7. Remove Exact duplicates 🡪 Yes
8. Ignore feature strand information 🡪 No
9. Make Probes 🡪 Over Feature +/-500 bp
10. Identifies **191,857** probes 🡪 Name “ROCs”
11. Add ROCs to annotation track (Right click probe list, select “Convert to annotation track”)
12. Filter ROCs that were are 1kbp of Refseq curated genes (Filtering 🡪 Filter by features)
    - 1. Features to design around – RefSeq_Curated.bed
      2. Split into subfeatures – No
      3. Make Probes- Over feature +/-1000bp
      4. Select probes which are – overlapping
      5. Distance cutoff (bp) – n/a
      6. Use features on strand – Any
      7. Identifies 68,856 ROCs that are overlapping or within 1kb of RefSeq genes
13. Filter ROCs that were within 1kbp of UCSC genes (Filtering 🡪 Filter by features)
    - 1. Features to design around – UCSC_RefSeq.bed
      2. Split into subfeatures – No
      3. Make Probes- Over feature +/-1000bp
      4. Select probes which are – overlapping
      5. Distance cutoff (bp) – n/a
      6. Use features on strand – Any
      7. Identifies 71,461 ROCs that are overlapping or within 1kb of UCSC genes
14. Filter ROCs that within 1kbp of Ensemble genes (Filtering 🡪 Filter by features)
    - 1. Features to design around – Rn6_v95_gtf_genes.txt
      2. Split into subfeatures – No
      3. Make Probes- Over feature +/-1000bp
      4. Select probes which are – Overlapping
      5. Distance cutoff (bp) – n/a
      6. Use features on strand – Any
      7. Identifies 76,382 ROCs overlapping or within 1kb of Ensemble genes
15. Filtered ROCs that overlap Contiguously transcribed regions (Filtering 🡪 Filter by features)
    - 1. Features to design around – Contigs.txt
      2. Split into subfeatures – No
      3. Make Probes- Over feature +/-1000bp
      4. Select probes which are – Overlapping
      5. Distance cutoff (bp) – n/a
      6. Use features on strand – Any
      7. Identifies 57366 ROCs overlapping or within 1kb of Contiguously transcribed regions
16. Repeat filtering for miRNA, misc_RNA, rRNA, snoRNA, snRNA, tRNA (Filtering 🡪 Filter by features)
    - 1. Features to design around – pick from above annotations provided by Seqmonk Rn6 genome assembly
      2. Split into subfeatures – No
      3. Make Probes- Over feature From +/- 0bp
      4. Select probes which are – Overlapping
      5. Distance cutoff (bp) – n/a
      6. Use features on strand – Any
      7. 839 ROCs overlapping various ncRNA genes
17. Filtering 🡪 intersect multiple lists…
    1. Include ROCs, exclude all ROCs overlapping features from sections 4-8 of Step 6
    2. Intersection leaves **100,767** “Intergenic ROCs” or iROCs
    3. Convert to annotation track (Right click probe list, select “Convert to annotation track”)

**Steps 7-8.** Using Seqmonk, quantify total RNA-seq transcription from iROCs. Then, filter for iROCs that are bidirectionally transcribed.

1. Design probes around iROCs (Data 🡪 Define Probes 🡪 Feature Probe Generator)
   1. Features to design around 🡪 iROCs
   2. Split into subfeatures 🡪 No
   3. Remove Exact duplicates 🡪 Checked
   4. Ignore feature strand information 🡪 Unchecked
   5. Make Probes 🡪 Over Feature from -0 to +0 bp
2. Quantify all sequenced transcripts (Probe quantitation 🡪 Difference Quantitation)
   - 1. Calculate Forward only as a percentage of All reads
     2. Min count = 0
     3. Ignore duplicates – unchecked
3. Filter unidirectionally transcribed iROCs (Filtering 🡪 Filter on Values)
   1. Merged RNA-seq group value must be between 5 and 95%
   2. This excludes probes with unidirectional transcription from either strand (likely unannotated genes or exons)
   3. Identifies **28,492** “Transcriptionally active putative enhancers” – TAPEs
   4. Convert to annotation track Right click probe list, select “Convert to annotation track”
4. Make probes defining TAPEs (Define probes 🡪 Feature Probe Generator)
   1. Features to design around 🡪 “TAPEs”
   2. Split into subfeatures 🡪 No
   3. Remove exact duplicates 🡪 Checked
   4. Ignore strand feature information 🡪 Unchecked
   5. Make probes 🡪 Over feature -0 to +0 bp
5. Quantify TAPE transcription (CPKM) (Data 🡪 Read count quantitation)
6. Count reads on strand 🡪 All reads
7. Correct for total read count 🡪 Checked
8. Correct to what? 🡪 per million reads
9. Count total only in probes? 🡪 Unchecked
10. Correct for probe length? 🡪 Checked
11. Log transform count 🡪 Unchecked
12. Count duplicate reads only once 🡪 Unchecked
13. Filtering 🡪 Probe Values filter
14. RNA-seq group value must be between 0.05 and 10000
15. Identifies 1916 highly expressed TAPEs
16. Create report for TAPEs
17. Reports 🡪 Annotated Probe Report
18. Annotate with overlapping Rn6_v95_gtf_genes.txt
19. Annotation distance cutoff – 1Mbp
20. Include – unannotated probes
21. Include – data for currently visible stores

**Steps 9.** Using R, identify all genes +/- 1Mbp of TAPEs. Then, correlate transcription at each TAPE with transcription at each gene.

# Mapping Transcriptionally Active Putative Enhancers (TAPEs) to Genes

The goal of this analysis is to identify high confidence TAPE-gene pairs. To do this, transcription start sites located within 1Mb upstream or downstream from the center of the TAPE are identified. Then, pearson’s correlations are calculated using counts for the TAPEs and associated genes

## Load Libraries

First, all essential libraries are loaded for the analysis.

suppressPackageStartupMessages(library(dplyr))
suppressPackageStartupMessages(library(Seurat))

## Warning: package 'Seurat' was built under R version 3.6.2

suppressPackageStartupMessages(library(ggplot2))
suppressPackageStartupMessages(library(cowplot))
suppressPackageStartupMessages(library(data.table))
suppressPackageStartupMessages(library(VennDiagram))

## Load in Required Data

First, read in the 28,492 TAPEs identified in the **Step 8.**

TAPEs <- read.table(file = "~/TAPE_RNA_quantification_CPKM.txt",sep = "\t",header = TRUE)

Now we are going to create a column indicating these as intergenic TAPEs. Then, we are going to create an ID column consisting of the TAPE chromosome, starting position, and ending position. Next, we will rename the first four columnns. The start and end will be referred to as five and three prime end as the SeqMonk software used during the pipeline refers to the 5’ end of all genes as the start, regardless of whether that gene is on the - strand. Thus, referring to the start/end as five prime and three prime will allow us to correctly calculate distance later in this workflow. Finally, the center of the TAPE is calculated.

#Give a column indicating the type
TAPEs$Type <- "Intergenic"

#Create a column with a unique ID.
TAPEs$ID <- paste(TAPEs$Chromosome,TAPEs$Start,TAPEs$End,sep = "_")

#Change the TAPEs names
names(TAPEs)[1:4] <- c("TAPE_ID","TAPE_Chr","TAPE_Five_Prime_End","TAPE_Three_Prime_End")

#Calculate the center of the TAPE
TAPEs$TAPE_Center <- TAPEs[,"TAPE_Five_Prime_End"] + (round(abs(TAPEs[,"TAPE_Five_Prime_End"] - TAPEs[,"TAPE_Three_Prime_End"])/2))

Next, read in the genes used during TAPE identification and change the column names.

#read in the genes that were used to during enhancer identificaiton
genes <- read.delim(file = "~/Rn6_v95_gtf_genesonly_noBS.txt",sep = "\t")

#Change column naems
names(genes) <- c("Chr","Gene_Five_Prime_End","Gene_Three_Prime_End","Strand","Gene")

To map the TAPEs to associated genes, we will use a for loop. As the for loop proceeds, any TAPE that maps to the gene will be input into a large list created below. Here, every element of the list corresponds to a unique gene.

# Here I make an empty list to input the annotated TAEs into
genes_list <- vector(mode = "list",
 length = nrow(genes))
#Name every element of the list a unique name for that gene. This consists of chr,5' end, 3' end, Gene name, and strand that is comma separated
names(genes_list) <- paste(paste(paste(paste(genes$Chr,genes$Gene_Five_Prime_End,sep = ","),
 genes$Gene_Three_Prime_End,sep = ","),
 genes$Gene,sep = ","),
 genes$Strand,sep = ",")

Next we run a sanity check to make sure that the dataframe and list are in the same order.

#Make sure the dataframe and list are in the same order
all(as.character(lapply(strsplit(names(genes_list),split = ","),"[",4)) == genes$Gene) # TRUE

## [1] TRUE

all(as.character(lapply(strsplit(names(genes_list),split = ","),"[",5)) == genes$Strand) # TRUE

## [1] TRUE

all(as.character(lapply(strsplit(names(genes_list),split = ","),"[",1)) == genes$Chr) # TRUE

## [1] TRUE

all(as.numeric(lapply(strsplit(names(genes_list),split = ","),"[",2)) == genes$Gene_Five_Prime_End) # TRUE

## [1] TRUE

all(as.numeric(lapply(strsplit(names(genes_list),split = ","),"[",3)) == genes$Gene_Three_Prime_End) # TRUE

## [1] TRUE

# Identify TAPE-Gene Pairs

Now we annotate all genes that are 1Mbp upstream and downstream of the TAPE. This loops through the genes dataframe, and first asks if the strand of the gene is + or -. If the strand of the gene is positive all calculations are computed using the five prime end of the gene. If the strand of the gene is negative all calculations are computed using the three prime end of the gene. This search is gene-centric in that this loop identifies genes that fall within 1Mbp windows from the center of the TAPE.A progress bar will also print the progress of the loop.

Next, we identify any genes in which there were no associated TAPEs. These empty elements are then removed from the list.

#Empty vector for identification of empty list elements
x <-vector()
#Run loop
for(i in 1:length(genes_list)){
 if(is.null(genes_list[[i]])){
 x <- append(x = x,i)
 }else{
 next
 }
}
#Remove genes with no TAPEs
genes_list <- genes_list[-x]

This for loop adds a column to every dataframe in the list that indicates the gene name, strand, chr, 5’end, 3’ end. The list is then unlisted to create a large dataframe that can be exported. Finally, a sanity check is run to make sure that no rows are duplicated.

for(i in 1:length(genes_list)){
 genes_list[[i]]$Gene <- as.character(lapply(strsplit(names(genes_list)[i],split = ","),"[",4))
 genes_list[[i]]$Strand <- as.character(lapply(strsplit(names(genes_list)[i],split = ","),"[",5))
 genes_list[[i]]$Gene_Chr <- as.character(lapply(strsplit(names(genes_list)[i],split = ","),"[",1))
 genes_list[[i]]$Gene_Five_Prime_End <- as.numeric(lapply(strsplit(names(genes_list)[i],split = ","),"[",2))
 genes_list[[i]]$Gene_Three_Prime_End <- as.numeric(lapply(strsplit(names(genes_list)[i],split = ","),"[",3))
}

#unlist and make a huge dataframe
TAPEs_df <- rbindlist(genes_list)

#There should not be any duplicated rows, but this command is a sanity check
TAPEs_df <- as.data.frame(distinct(TAPEs_df)) #433,416

Here distance is calculated. If the gene is on the + strand, the TSS is the five prime end of the gene. If the gene is on the - strand, the TSS is the three prime end of the gene.

# Calculate distance and orientation based on strand
TAPEs_df$Distance <- NA
TAPEs_df$Orientation <- NA
TAPEs_df$Orientation <- as.character(TAPEs_df$Orientation)
#Calculate distance and orientation based on strand
#To calculate the distance, the TSS for each gene must be identified. The TSS changes for each gene's strandedness in that a + stand gene's TSS will be in the Gene_Five_Prime_End column and a - strand gene's TSS will be in the Gene_Three_Prime End column
TAPEs_df$TSS <- NA
TAPEs_df <- TAPEs_df %>% mutate(TSS = ifelse(Strand == "+",
 TAPEs_df$Gene_Five_Prime_End,
 TAPEs_df$Gene_Three_Prime_End))

Next, the orientation of the gene to the TAPE is identified and distance from center of TAPE to TSS of gene is calculated.

# #If the Gene's TSS is < the TAPE center and > TAPE-1e6 then the gene is upstream of the enhancer
# #If the Gene's TSS is > the TAPE center and < TAPE+1e6 then the gene is downstream of the enhancer
TAPEs_df <- TAPEs_df %>% mutate(Orientation = ifelse((TAPEs_df$TSS < TAPEs_df$TAPE_Center) & (TAPEs_df$TSS > (TAPEs_df$TAPE_Center - 1e+06)),
 "Upstream",
 ifelse((TAPEs_df$TSS > TAPEs_df$TAPE_Center) & (TAPEs_df$TSS < (TAPEs_df$TAPE_Center+1e+06)),
 "Downstream",
 0)
)
)
#Calculate Distance
TAPEs_df <- TAPEs_df %>% mutate(Distance = ifelse(Orientation == "Upstream",
 TAPEs_df$TAPE_Center - TAPEs_df$TSS,
 TAPEs_df$TSS - TAPEs_df$TAPE_Center))
#Another sanity check that the chromosomes of the TAPEs and the genes are the same
table(as.character(TAPEs_df$TAPE_Chr) == as.character(TAPEs_df$Gene_Chr))

##
## TRUE
## 433416

To calculate correlations we need TAPE and gene counts. The TAPE counts are already within the TAPEs dataframe. Here, we load in mRNA count information. Next, we keep only genes in which there is one annotation. Finally, only useful columns are kept.

#Read in the Gene Probe counts identified with Seqmonk
Gene_Probe_Counts <- read.delim(file = "~/mRNA_quantification_CPKM.txt",sep = "\t")
#Keep only genes in which there is one annotated gene.
Gene_Probe_Counts <- Gene_Probe_Counts[!duplicated(as.character(Gene_Probe_Counts$Probe)),]
#Pull out useful columns Probe, Sample Counts
Gene_Probe_Counts <- Gene_Probe_Counts[,c(1,13:31)]

Within this loop, Pearson’s correlations are calculated.

#Make columns for correlations
TAPEs_df$Cortex_Correlation <- NA
TAPEs_df$Hippocampus_Correlation <- NA
TAPEs_df$Striatum_Correlation <- NA
TAPEs_df$Global_Correlation <- NA
x <- vector()
#Now calculate correlations
for(i in 1:nrow(TAPEs_df)){
 #figure out which row in the Gene_Probe_Counts column corresponds to the gene in the TAPEs_df column
 row <- which(as.character(TAPEs_df[i,"Gene"]) == as.character(Gene_Probe_Counts$Probe))
 if(length(row) >0){
 #####Cortex####
 #Calculate the correlation
 TAPEs_df[i,"Cortex_Correlation"] <- cor(y = as.numeric(TAPEs_df[i,c(13:18)]),x = as.numeric(Gene_Probe_Counts[row,c(2:7)]))
 #####Hippocampus######
 #Calculate the correlation
 TAPEs_df[i,"Hippocampus_Correlation"] <- cor(y = as.numeric(TAPEs_df[i,c(19:24)]),x = as.numeric(Gene_Probe_Counts[row,c(8:13)]))
 #######Striatum#######
 #Calculate the correlation
 TAPEs_df[i,"Striatum_Correlation"] <- cor(y = as.numeric(TAPEs_df[i,c(25:31)]),x = as.numeric(Gene_Probe_Counts[row,c(14:20)]))
 #Gloabl Correlation
 #calculate the correlation
 TAPEs_df[i,"Global_Correlation"] <- cor(y = as.numeric(TAPEs_df[i,13:31]),x = as.numeric(Gene_Probe_Counts[row,2:20]))
 }else{
 x <- append(x = x,i)
 }
}

Some of the genes have count values of 0 for every sample across all cell types. This results in a correaltion value of 0. Thus, these TAPE-gene pairs are removed, leaving us with 388,605 potential TAPE-gene pairs.

TAPEs_df <- TAPEs_df[!is.na(TAPEs_df$Global_Correlation),]
nrow(TAPEs_df) #388,605

## [1] 388605
